# Neuroendocrine transcriptional programs adapt dynamically to the supply and demand for neuropeptides as revealed in *NSF *mutant zebrafish

**DOI:** 10.1186/1749-8104-4-22

**Published:** 2009-06-23

**Authors:** Deborah M Kurrasch, Linda M Nevin, Jinny S Wong, Herwig Baier, Holly A Ingraham

**Affiliations:** 1 Department of Cellular and Molecular Pharmacology, University of California, San Francisco, California, USA; 2 Department of Physiology, University of California, San Francisco, California, USA; 3 Gladstone Institute of Cardiovascular Disease, San Francisco, California 94158, USA

## Abstract

**Background:**

Regulated secretion of specialized neuropeptides in the vertebrate neuroendocrine system is critical for ensuring physiological homeostasis. Expression of these cell-specific peptide markers in the differentiating hypothalamus commences prior to birth, often predating the physiological demand for secreted neuropeptides. The conserved function and spatial expression of hypothalamic peptides in vertebrates prompted us to search for critical neuroendocrine genes in newly hatched zebrafish larvae.

**Results:**

We screened mutant 5 days post-fertilization zebrafish larvae that fail to undergo visually mediated background adaptation for disruption in hypothalamic *pomc *expression. To our surprise, the ATPase *N-ethylmaleimide sensitive factor *(*nsf*) was identified as an essential gene for maintenance of neuroendocrine transcriptional programs during the embryo-to-larva transition. Despite normal hypothalamic development in *nsf*^*st*53 ^mutants, neuropeptidergic cells exhibited a dramatic loss of cell-specific markers by 5 days post-fertilization that is accompanied by elevated intracellular neuropeptide protein. Consistent with the role of NSF in vesicle-membrane fusion events and intracellular trafficking, cytoplasmic endoplasmic reticulum-like membranes accumulate in *nsf*^-/- ^hypothalamic neurons similar to that observed for *SEC18 *(*nsf ortholog*) yeast mutants. Our data support a model in which unspent neuropeptide cargo feedbacks to extinguish transcription in neuropeptidergic cells just as they become functionally required. In support of this model we found that *gnrh3 *transcripts remained unchanged in pre-migratory, non-functional gonadotropin-releasing hormone (GnRH) neurons in *nsf*^-/- ^zebrafish. Furthermore, *oxytocin-like *(*oxtl*, *intp*) transcripts, which are found in osmoreceptive neurons and persist in mutant zebrafish, drop precipitously after mutant zebrafish are acutely challenged with high salt.

**Conclusion:**

Our analyses of *nsf *mutant zebrafish reveal an unexpected role for NSF in hypothalamic development, with mutant 5 days post-fertilization larvae exhibiting a stage-dependent loss of neuroendocrine transcripts and a corresponding accumulation of neuropeptides in the soma. Based on our collective findings, we speculate that neuroendocrine transcriptional programs adapt dynamically to both the supply and demand for neuropeptides to ensure adequate homeostatic responses.

## Background

The hypothalamus participates in the maintenance of homeostasis through the synthesis and release of neuropeptides. The neuroendocrine system appears to be highly conserved between mammals and teleosts, as evidenced by shared programs found for pituitary development [1-4] and the spatial patterns and functional roles of hypothalamic neuropeptides [5-11]. In teleosts, much of the neuroendocrine system becomes functional between 48 and 72 hours post-fertilization (hpf), when the embryo hatches from its chorion and becomes fully exposed to an external environment [12]. By 5 days post-fertilization (dpf) this newly hatched embryo has developed into a free-swimming larva that must respond and adapt to external and internal cues. Thus, this embryo-to-larva transition (3 to 5 dpf) is a distinct developmental stage, somewhat analogous to mammalian birth when the newborn integrates sensory input and initiates regulated-secretion to maintain homeostasis.

While the demand for neuroendocrine signaling and regulated neuropeptide secretion increases dramatically when a newborn is exposed to an external environment, cell-specific transcriptional programs appear to initiate much earlier [13-16]. Indeed, birthdating experiments in rodents show that hypothalamic neurons originate from the third ventricular neuroepithelium beginning at embryonic day (E)12 and are largely differentiated by E16 [17,18], as judged by expression and translation of cell type-specific neuropeptide transcripts and protein, respectively. That the onset of hypothalamic transcriptional programs predates function is perhaps best illustrated by the ontogeny of gonadotropin-releasing hormone (GnRH) neurons (also known as luteinizing hormone-releasing hormone (LHRH) neurons). In both mammals and zebrafish this subpopulation of neurons migrates from the olfactory placode into the hypothalamus and eventually orchestrates germ cell maturation at the onset of sexual maturity [6,19-23]. However, during embryonic development and prior to their migration, immature GnRH neurons express their signature neuropeptide [6,21,22]. These and other studies suggest that the neuroendocrine system develops early in the fetus and remains primed to secrete neuropeptides at later time points [24,25].

To identify genes that might be critical for hypothalamic development during this embryo-to-larva transition, we re-screened mutant 5 dpf zebrafish larvae that fail to undergo visually mediated background adaptation (VBA) for disruption in neuropeptide transcription. VBA is a well characterized neuroendocrine reflex that occurs in fish, reptiles, and amphibians (reviewed in [26]). When placed on a dark, light-absorbing background, pituitary melanotropes release α-melanocyte-stimulating hormone; this neuropeptide mediates dispersion of black pigment granules in dermal melanophores and causes a darkening of skin color in fish. Conversely, when placed on a light background, a drop in α-melanocyte-stimulating hormone levels results in the aggregation of melanin and a loss of this 'dark' phenotype or an overall blanching. Because α-melanocyte-stimulating hormone is directly regulated by hypothalamic inputs, we reasoned that critical developmental neuroendocrine genes might be discovered in VBA-mutant zebrafish [27] with altered *proopiomelanocortin *(*pomc*) expression.

From our screen, one mutant line showed a complete and specific loss of *pomc *expression in the presumptive arcuate nucleus of the hypothalamus. To our surprise, this mutated gene was identified as *N-ethylmaleimide sensitive factor *(*nsf*, *SEC18*), which is an ATPase and functions in membrane fusion events as well as acting as a structural chaperone (reviewed in [28,29]). These functional roles of NSF are important for regulated secretion [30-32] and for intracellular trafficking [33]. Our analysis of *nsf *mutant zebrafish led us to examine how hypothalamic developmental programs respond to a major secretory defect. We report that *nsf*-deficient hypothalamic neurons exhibit a marked accumulation of neuropeptide proteins with a corresponding stage-dependent loss of mRNA, suggesting that neuropeptide stores in the cytoplasm feedback to regulate transcriptional programs in the nucleus.

## Results

### Loss of hypothalamic *pomc *expression in *nsf *mutant zebrafish

**Figure 1:**
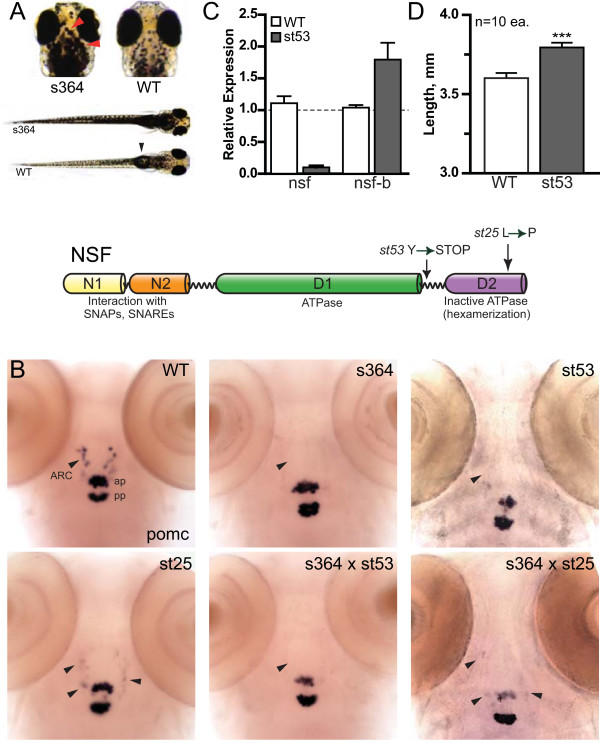
**The s364 phenotype is due to mutation in *nsf***. **(A) **Mutant and wild-type (WT) zebrafish (5 dpf) are shown on a white background. Mutant zebrafish display a dark appearance on the white background from dispersed melanin (red arrowhead). Wild-type zebrafish have an inflated swim bladder (black arrowhead) but mutant zebrafish do not. **(B) **Loss of *pomc *expression in the arcuate nucleus (ARC) of the hypothalamus (arrowheads) of mutant alleles but not wild-type at 5 dpf is shown. *pomc *expression is maintained in the anterior pituitary (ap) and posterior pituitary (pp) in both wild-type and mutant alleles. *s364*/*st53 *or *s364*/*st25 *transheterozygotes display the same *pomc *phenotype as *s364*, *st53*, and *st25 *homozygotes. Images are representative of at least three independent crosses of fifteen or more fish each. **(C) **Relative transcript levels are shown for *nsf *and *nsfb *in wild-type and *st53 *zebrafish heads at 5 dpf (n = 8 each). Fold induction is relative to one wild-type head for each primer set and taken to be 1.0 (horizontal dashed line). All other samples, including additional control and experimental samples, are normalized to this one wild-type sample with variation shown by error bars (standard error of the mean). **(D) **The linear growth of 5 dpf wild-type or *st53 *zebrafish is shown (wild-type = 3.6 mm, *st53 *= 3.8 mm; n = 10 each group). Length was measured from nose to tip of tail. Wild-type fish were temporarily paralyzed using ice. Results are expressed as mean ± standard deviation, and statistical analyses were done by unpaired *t*-test. ***P < 0.001. A schematic representation of NSF protein motifs. The *st53 *mutation introduces a nonsense mutation just prior to D2, whereas *st25 *introduces a missense mutation in D2. Two distinct amino-terminal domains (N1, N2) mediate substrate recognition.

To identify genes critical to neurosecretory cell function, we screened a collection of 50 VBA-deficient zebrafish mutants for disrupted *pomc *expression. These mutants have been described previously in a forward genetic screen employing the chemical mutagen ethyl-nitroso urea [27]. Five mutants with a range of hypothalamic defects were identified in our screen, one of which (*s364*) is reported here. *s364 *mutant zebrafish display a dark appearance on light background and lack an inflated swim bladder (Figure 1A). Additionally, mutant zebrafish have a listless startle response, become paralyzed, and eventually die by 8 dpf (data not shown). The mutation was mapped to chromosome 3 of the zebrafish genome using polymorphic microsatellite markers (HB, unpublished data). This position matched that of the highly conserved AAA ATPase *nsf*, for which two alleles (*st25 *and *st53*) have been identified previously in an unrelated screen [34]. The *st25 *and *st53 *alleles harbor nonsense and missense *nsf *mutations carboxy-terminal to the ATPase domain (D1) and in the oligomerization domain (D2), respectively [34] (Figure 1). The *s364 *and previously reported *nsf *mutants [34] exhibit similar locomotor and VBA phenotypes.

Multiple complementation crosses revealed that *s364 *represents a novel loss-of-function allele of *nsf*. Similar to *s364*, both *st53 *and the *s364*/*st53 *and *s364*/*st25 *transheterozygotes showed disrupted *pomc *staining in the arcuate nucleus of the hypothalamus in mutant zebrafish, whereas expression was maintained in the anterior and posterior pituitary (Figure 1B). Low, but persistent *pomc *expression was observed in the arcuate nucleus of the missense *nsf *allele *st25 *and in the *s364*/*st25 *transheterozygotes (Figure 1B). Sequencing of the exons and the exon-intron junctions of *s364 *revealed no obvious mutations, suggesting that this *nsf *allele contains a non-coding mutation (data not shown). Because the genetic lesion generating the *s364 *allele remains unknown, we used *nsf*^*st*53 ^mutant zebrafish for all subsequent analyses so that mutant embryos could be identified by genotyping, prior to the appearance of the 'dark' phenotype.

### Disruption of *nsf *is partially compensated by upregulation of *nsfb*

The zebrafish genome contains duplicates of an ancestral *nsf *gene, annotated as *nsf *and *nsfb *[34]. *nsf *is ubiquitously expressed in the central nervous system throughout development [34] and in early larval stages (Additional file 1 Figure S3A). As might be predicted by the nonsense mutation in *nsf*^*st*53^, *nsf *transcript levels are nearly absent in *nsf*^*st*53 ^zebrafish (Figure 1C). However, quantitative PCR revealed that *nsfb *is upregulated nearly two-fold in the heads of *nsf *mutant zebrafish (Figure 1C). Thus, compensation by *nsfb *may account for the survival of *nsf*^*st*53 ^zebrafish to 8 dpf given that combined *nsf *and *nsfb *morpholino injections result in prominent and early lethality (Additional file 1 Table S1). Interestingly, *nsf*^*st*53 ^zebrafish were noted to be longer than wild-type siblings (3.8 mm versus 3.6 mm; Figure 1D), and while the molecular basis of this phenotype is unclear, others have found that disruption of POMC-melanocortin signaling increases linear growth in both zebrafish and mice [35,36].

### Hypothalamic cell markers are absent in *nsf*^*st*53 ^mutant larvae

**Figure 2:**
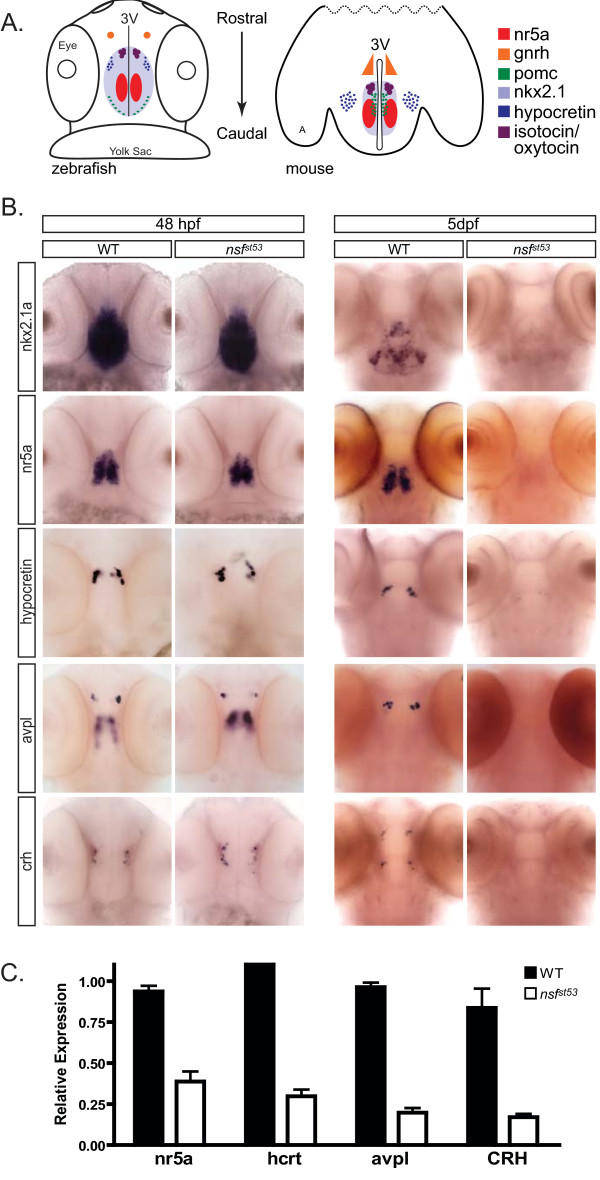
**Loss of hypothalamic markers occurs during embryo-to-larva transition**. **(A) **Cartoon depicting the spatial patterning of known hypothalamic markers in the zebrafish (48 hpf) and mouse (P0) brain. A dorsal view, ventral side down is shown for zebrafish and a horizontal plane is shown for mouse of several conserved hypothalamic transcription factors and neuropeptides. Other anatomical landmarks include the eyes, yolk sac, third ventricle (3V), and on the mouse cartoon, amygdala (A), **(B) **Whole mount expression patterns are shown for *nkx2.1a*, *nr5a *(*ff1d*), *hcrt*, *avpl*, and *crh *for both 48-hpf (n = 15) and 5-dpf (n = 20) wild-type (WT) and *nsf*^*st*53 ^zebrafish. The expression of hypothalamic markers is lost by 5 dpf (right panel). The 48-hpf *nsf*^*st*53 ^zebrafish were identified by genotyping. **(C) **Relative transcript levels of neuroendocrine markers are shown in wild-type and *nsf*^*st*53 ^zebrafish heads at 5 dpf (n = 8 each). Fold induction is relative to 1.0 with results from a single wild-type head chosen for each primer set. Samples variation is shown by error bars (standard error of the mean).

**Figure 3:**
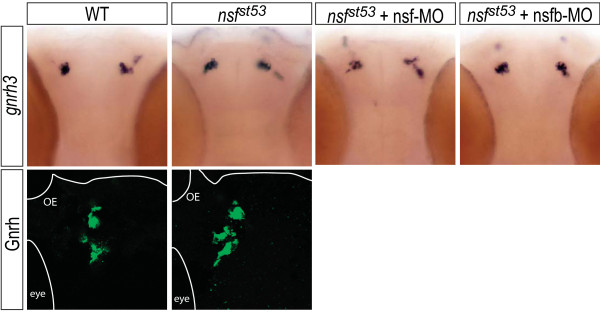
***gnrh3 *transcripts persist and protein levels are normal in *nsf*^*st*53 ^zebrafish**. Whole-mount *in situ *hybridization for *gnrh3 *expression in the olfactory placode in 5 dpf wild-type (WT) and *nsf*^*st*53 ^control or morpholino-injected larva is shown (top panels). Immunocytochemistry for GnRH peptide levels is shown (bottom panels). Images are representative of at least four animals per genotype per treatment. Anatomical landmarks include the olfactory epithelium (OE) and the eye.

We next determined if loss of *pomc *expression in *nsf*^*st*53 ^zebrafish extended to other hypothalamic markers, including transcription factors and neuropeptides that are highly conserved between teleosts and mammals (Figure 2A). Indeed, nearly all hypothalamic transcripts were lost by 5 dpf in *nsf*^*st*53 ^zebrafish (Figure 2B, C; Additional file 1 Figure S5). Loss of hypothalamic markers in these secretory-defective zebrafish was stage-dependent as evidenced by the normal expression observed at earlier developmental time points (24 hpf; data not shown) and just prior to hatching (48 hpf; Figure 2B). A time course through the embryo-to-larva transition revealed that expression of these hypothalamic markers begins to decrease at 4 dpf, and is completely absent by 5 dpf in *nsf*^*st*53 ^zebrafish (Figure 2B, C; Additional file 1 Figure S1). These data suggest that the neuroendocrine system in *nsf*^*st*53 ^zebrafish undergoes normal development but loses differentiated cell markers between 3 and 5 dpf. In contrast, expression of *emx1 *in the forebrain (Additional file 1 Figure S2), *pomc *in the pituitary (Figure 1B), and *gnrh3 *in the nasal region (Figure 3) are all maintained at normal levels, suggesting that this *nsf *mutation selectively reduced expression of transcripts in the hypothalamus proper.

### Loss of hypothalamic markers is not due to apoptosis

**Figure 4:**
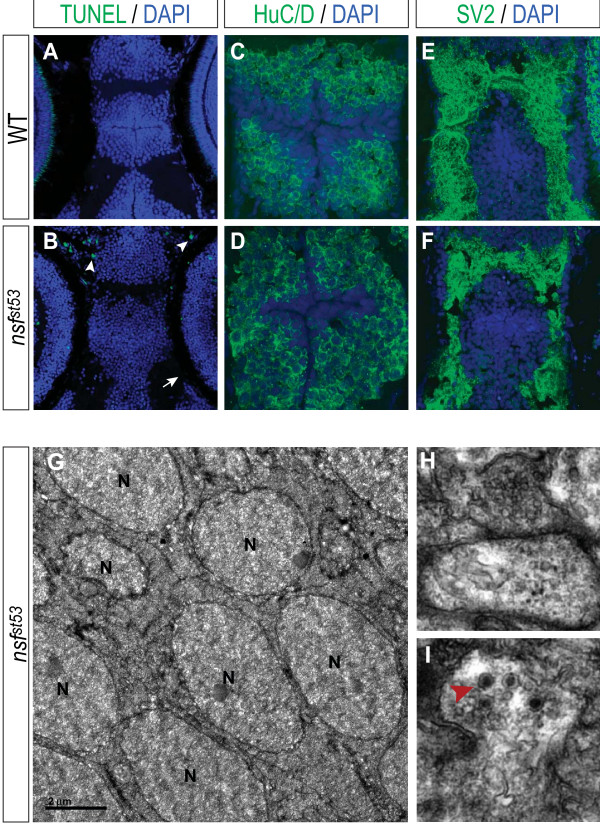
***nsf*^*st*53 ^hypothalamus undergoes normal development and does not appear to initiate apoptosis**. Apoptosis in **(A) **wild-type (WT) and **(B) ***nsf*^*st*53 ^5 dpf zebrafish (12 μm horizontal sections) is shown. Cell death is not observed in the hypothalamus (B), but is observed in olfactory terminals (B, white arrowhead). Photoreceptor auto-fluorescence in wild-type zebrafish is lost in mutant (B, white arrow). DAPI staining of wild-type (A, C, E) and *nsf*^*st*53 ^zebrafish (B, D, F) at 5 dpf is shown. Immunocytochemistry for **(C, D) **Hu family proteins and **(E, F) **SV2 are shown for wild-type and *nsf*^*st*53 ^5 dpf zebrafish. All images are representative of at least 15 animals assayed. **(G-I) **Electron microscopy images from *nsf*^*st*53 ^hypothalamic sections (n = 3). Cell bodies (G) and vesicle-filled synapse (H) resemble wild-type neurons (data not shown). Anatomical landmarks and (I) the presence of dark-cored secretory vesicles (red arrowhead) were used to confirm the location of the hypothalamus. N = nucleus. Note that the medially located cell bodies (Figure 4C, D, G) are framed on either side by lateral projections (Figure 4E, F, H) in both cryosection and EM images.

The comprehensive loss of neuroendocrine markers at 5 dpf in *nsf*^*st*53 ^zebrafish was not explained by obvious defects in gross anatomy of the hypothalamus or surrounding areas (Figure 4A, B, G-I). For example, DAPI staining showed a distinct and well-formed hypothalamus in *nsf*^*st*53 ^zebrafish without any obvious evidence of nuclear condensation or apoptosis (Figure 4A, B; Additional file 1 Figure S4A, B). In fact, TUNEL (terminal deoxynucleotidyltransferase-mediated dUTP nick end labeling) staining on sections (Figure 4B) and whole-mounted (Additional file 1 Figure S3B) *nsf*^*st*53 ^zebrafish fixed at 12 h intervals between 48 hpf through 5.5 dpf failed to reveal hypothalamic cell death at all time points. On the other hand, in *nsf*^*st*53 ^mutants one easily observes classic hallmarks of cell death in the hindbrain (Additional file 1 Figure S4C, D) and in several sensory neuronal populations such as the olfactory neurons (Figure 4B; Additional file 1 Figure S4F), otic neurons (Additional file 1 Figure S4H), and photoreceptors (Additional file 1 Figure S4J). Moreover, we found that *nsf*^*st*53 ^hypothalamic neurons differentiated and organized normally, as judged by staining for HuC/D (Figure 4C, D) and synaptic vesicle protein 2 (SV2; Figure 4E, F). The slight decrease in SV2 staining (Figure 4F) coupled with DiI tracing studies (data not shown) suggested that *nsf*^*st*53 ^neurons have lowered vesicle pools, but retain normal projections. More importantly, electron microscopy (EM) analysis confirmed that *nsf*^*st*53 ^hypothalamic neurons exhibit relatively normal morphology with evidence of synapse formation (Figure 4G, H). Taken together, our data suggest that *nsf *mutant neurosecretory cells are able to differentiate correctly and remain viable through early larval stages, but fail to maintain cell-specific transcriptional programs at later stages.

### Hypothalamic neuropeptides accumulate in *nsf*^*st*53 ^mutants

**Figure 5:**
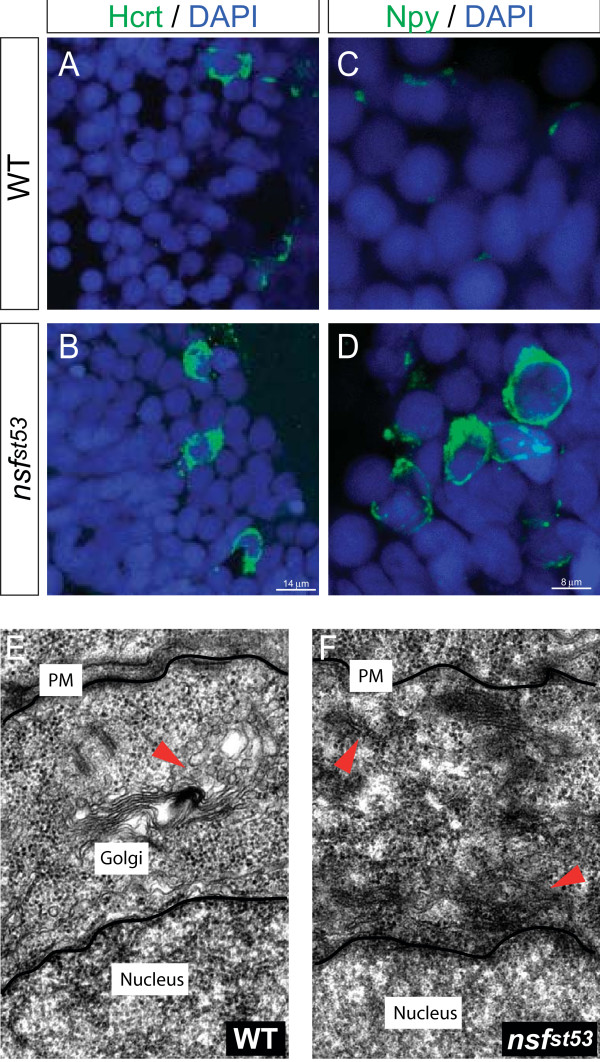
**Accumulated neuropeptide protein and the buildup of endoplasmic reticulum-like membranes in *nsf*^*st*53 ^zebrafish**. The levels of **(A, B) **hypocretin and **(C, D) **NPY protein in wild-type (WT) and *nsf*^*st*53 ^zebrafish are shown (n = 8 each). Protein levels are increased in the soma (B, D). DAPI stained nuclei are also shown (A-D). Electron microscopy images from **(E) **wild-type and **(F) ***nsf*^*st*53 ^zebrafish (n = 3 each). For EM analysis, neuroanatomical landmarks (obtained from [50]) and the presence of dense-core vesicles (Figure 4I) were used to locate the hypothalamus. The budding of secretory vesicles from the trans-Golgi network is highlighted (E, red arrowhead). A build up of ribosome-bound ER-like membranes in *nsf*^*st*53 ^zebrafish is noted (F, red arrowhead). PM = plasma membrane.

Having shown that transcriptional programs are dampened in neuroendocrine cells in *nsf*^*st*53 ^zebrafish, we next determined if protein levels were also diminished. Unexpectedly, 5 dpf *nsf*^*st*53 ^zebrafish exhibited prominent accumulation of hypocretin and neuropeptide Y (NPY) protein in the cell soma (Figure 5A–D; Additional file 1 Figure S5) with a concomitant decrease of neuropeptide in peripheral projections (Additional file 1 Figure S5). By contrast, only moderate (hypocretin) or faint (NPY) staining was observed in wild-type cell bodies (Figure 5A, C; Additional file 1 Figure S5) with higher protein levels observed in cell projections (Additional file 1 Figure S5; data not shown). We noted that levels of another medially expressed protein, tyrosine hydroxylase, were equivalent in wild-type and mutant hypothalami (Additional file 1 Figure S2E, F). Correlating with this finding, EM analysis showed increased cytoplasmic endoplasmic reticulum (ER)-like membranes in mutant hypothalamic neurons. These morphological structures are decorated with ribosomes, but lack other secretory organelles, such as the Golgi or mature secretory granules (Figure 5E, F). Although the exact identity of these ER-like membranes remains to be determined, their gross morphology is reminiscent of ER accumulation observed in mutant *SEC18 *yeast, suggesting that the initial steps in ER-to-golgi trafficking are blocked in *nsf *mutants.

### Transiently blocking secretion in wild-type zebrafish mimics the *nsf*^*st*53 ^phenotype

**Figure 6:**
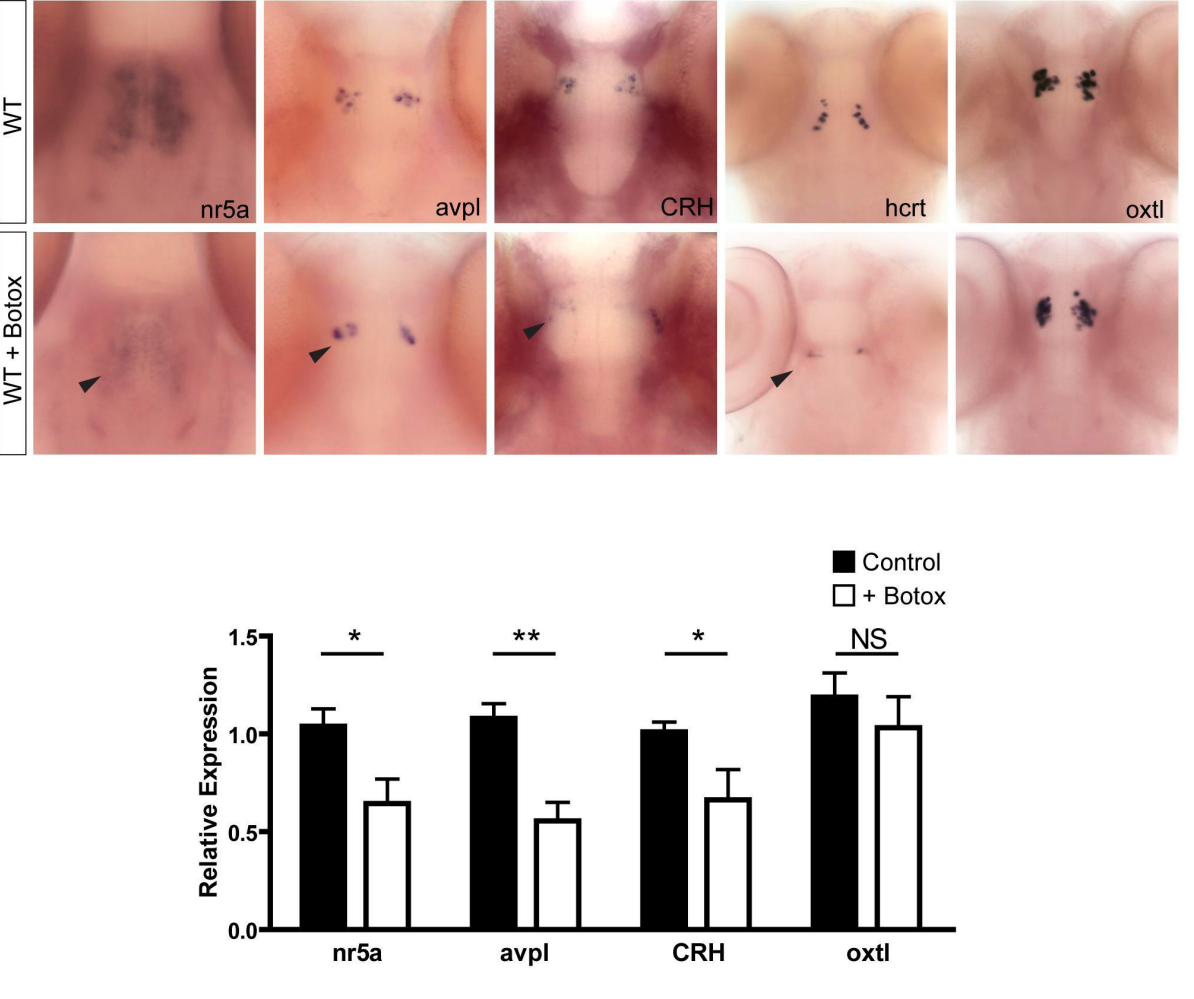
**Injection of botulinum toxin B into wild-type zebrafish mimics the *nsf*^*st*53 ^phenotype**. Whole-mount *in situ *hybridization for wild-type (WT) and WT + botox at 5 dpf is shown (n = 4 each). The expression of neuroendocrine markers in botox-injected WT fish is highlighted (black arrowheads). Relative transcript levels are shown for the heads of WT and WT + botox at 5 dpf (n = 8 each). Fold induction is relative to one control sample and taken to be 1.0. All other samples, including additional control and experimental samples, are normalized to this one wild-type sample. Sample variation is shown by error bars (standard error of the mean). Statistical analyses were done by unpaired *t*-test. *P < 0.05; **P < 0.01. NS = not significant.

We then asked if impairing general secretion in wild-type fish would induce a similar transcriptional dysregulation, as observed for the *nsf *mutants. Injections of botulinum toxin B, which prevents membrane fusion in the presynaptic plasma membrane [37] were performed at different doses (0.1 to 1 μg) into wild-type embryos at the one-cell stage. Zebrafish that were still paralyzed at 4 dpf, 5 dpf or 7 dpf were analyzed, and at all stages we noted a marked reduction in hypothalamic transcripts tested, except for oxytocin-like expression, which remained unchanged (Figure 6 and data not shown). These data bolster the notion that the *nsf*^*st*53 ^neuroendocrine phenotype arises from a general secretory defect.

### A physiological challenge silences persistent oxtl transcripts in *nsf*^*st*53 ^mutants

**Figure 7:**
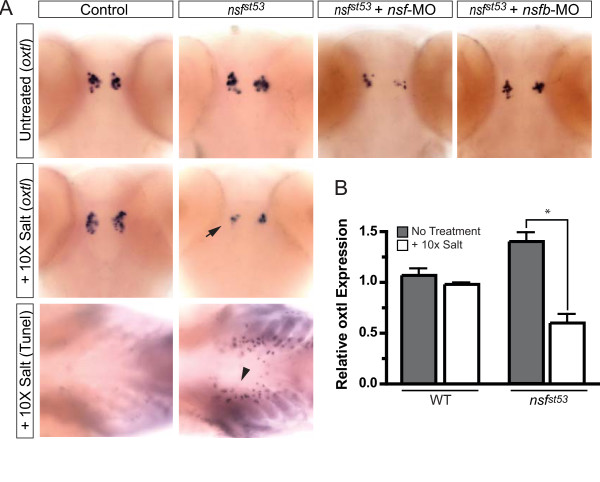
***oxytocin-like *is downregulated in *nsf*^*st*53 ^zebrafish following an acute osmolar challenge**. **(A) **Whole-mount *in situ *hybridization in 5 dpf wild-type (WT) and *nsf*^*st*53 ^control or morpholino-injected zebrafish (top panels) or following 24 h 10× salt treatment (middle panel) is shown. The decrease in expression of *oxytocin-like *(*oxtl*) in *nsf*^*st*53 ^zebrafish exposed to 10× salt is noted (black arrow). TUNEL-positive cells around the gills in *nsf*^*st*53 ^zebrafish following 10× salt treatment are shown (black arrowhead). Images are representative of at least four animals per genotype per treatment. The salt challenge was repeated twice. **(B) **Relative transcript levels are shown for *oxtl *in wild-type and *nsf*^*st*53 ^zebrafish heads at 5 dpf, with or without 10× salt exposure (n = 8 each). Fold induction is relative to one control sample and taken to be 1.0. All other samples, including additional control and experimental samples, are normalized to this one wild-type sample. Variation in samples is shown by error bars (standard error of the mean). Statistical analyses were done by unpaired *t*-test. *P < 0.05.

Of the nine hypothalamic markers tested, two neuropeptides were consistently maintained in 5 dpf *nsf*^*st*53 ^zebrafish – the zebrafish ortholog of oxytocin, *oxytocin-like *(*oxtl*; formerly known as isotocin [10]) and the GnRH isoform, *gnrh3*; both transcripts remained unchanged (Figures 3 and 7). For *gnrh3*, we also found that the corresponding protein levels were unaffected in mutant zebrafish (Figure 3). At this developmental stage *gnrh3*-expressing neurons are still immature and have not migrated from the olfactory placode to the medial hypothalamus to become fully functional [6]. Compensation by *nsfb *fails to account for the persistence of *oxtl *and *gnrh3*, with little or no reduction of either transcript observed after a dose-dependent knockdown of *nsfb *in mutant embryos using sequence-specific morpholino oligonucleotides (MOs; Figures 3 and 7). Control MO injections were without effect (data not shown). Interestingly, MO knock down of *nsf *resulted in a slight but noticeable reduction in *oxtl *and *gnrh3 *expression in *nsf*^*st*53 ^zebrafish (Figures 3 and 7), suggesting that maternally deposited *nsf *mRNA and/or a partially active truncated NSF protein accounts for full expression of *oxtl *and *gnrh3 *in the *nsf*^*st*53 ^allelic background.

We hypothesized that the selective loss of neuropeptide transcripts in *nsf*^*st*53 ^zebrafish might reflect activation of these particular neurosecretory cells during the embryo-to-larval transition. This 'use-dependent' transcriptional hypothesis predicts that *gnrh3 *and *oxtl *neurons are functionally silent at 3 to 5 dpf. If true, then activation of either *gnrh3 *or *oxtl *neurons during this developmental stage should diminish their respective neuropeptide transcripts. Although activating *gnrh3 *neurons is technically difficult, we reasoned that activating *oxtl *cells by a high-salt challenge might be feasible given that these neurons are osmoreceptive, and together with arginine vasopressin-like (*avpl; formerly known as vsnp*) neurons are required to maintain electrolyte homeostasis [38,39]. Indeed, we found *oxtl *expression significantly diminished after a hyperosmolar acute challenge in *nsf*^*st*53 ^zebrafish, but not in wild-type zebrafish (Figure 7A). This result was confirmed by quantitative PCR showing a nearly 50% reduction of *oxtl *transcripts after a 24 h salt challenge (Figure 7B). We also observed TUNEL-positive cells around the gills in salt-challenged *nsf*^*st*53 ^but not wild-type zebrafish (Figure 7A). Based on these observations, we suggest that the demand for oxytocin-like neuropeptide during a high salt challenge overloads the defective secretory pathway in *nsf*^*st*53 ^mutants, resulting in downregulation of *oxtl transcripts*.

## Discussion

**Figure 8:**
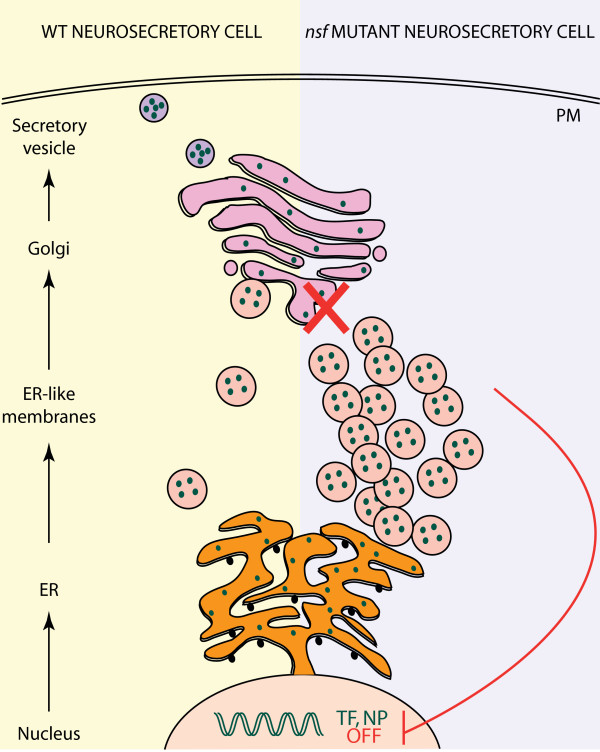
**Working model of *nsf*^*st*53 ^neuroendocrine cells**. A schematic of regulated neuropeptide secretion is shown, as adapted from [47,48]. In wild-type (WT) cells, newly translated neuropeptides in the endoplasmic reticulum (ER) are sorted in the trans-Golgi network, packaged into immature secretory granules, and finally released from mature dense-core secretory vesicles at the plasma membrane (reviewed in [49]). In secretion-defective *nsf*^*st*53 ^hypothalamic neurons, a buildup of ER-like membranes and soma accumulation of neuropeptides is observed. We hypothesize that in secretory-deficient *nsf *mutants, accumulation of 'unspent neuropeptide consumables' triggers a cytoplasmic-to-nuclear feedback signal that silences neuroendocrine transcriptional programs. NP = neuropeptide, PM = plasma membrane, TF = transcription factor.

Here, we unexpectedly identified *nsf *as essential for maintenance of cell type-specific programs in the zebrafish hypothalamus during a developmental transition when the demand for neuroendocrine signaling rises. We show that despite normal development in *nsf*^*st*53 ^hypothalami, neuropeptidergic cells exhibit a dramatic silencing of cell-specific markers by 5 dpf, with a concomitant accumulation of neuropeptides in the soma. Furthermore, while EM images show that *nsf*^*st*53 ^neuroendocrine cells appear normal in cell density, axonal growth, and synapse formation, these cells exhibit a build-up of cytoplasmic ER-like membranes consistent with the marked cytoplasmic accumulation of hypocretin and NPY neuropeptides in mutant hypothalami. Taken together, we propose that neuroendocrine transcription is normally modulated by cytoplasm-to-nucleus feedback signaling, which becomes exaggerated in the secretion-defective *nsf *mutant, as depicted in Figure 8.

Our results show that loss of hypothalamic cell markers occurs in all *nsf *alleles examined, well after neuroendocrine neurons initiate expression of their signature markers. Indeed, expression patterns of all assayed neuropeptides and transcription factors were indistinguishable in wild-type and *nsf*^*st*53 ^zebrafish, beginning as early as 24 hpf; a similar phenotype was also noted in the *nsf*^s364 ^allele (data not shown). This is surprising considering the general role of NSF in membrane fusion and its ubiquitous expression throughout development, and given that *Drosophila dNSF1 *mutants exhibit impaired neurogenesis [40]. It is possible that normal hypothalamic development in *nsf*^*st*53 ^may result from compensation by *nsfb*, maternally deposited *nsf *transcripts or residual activity of NSF encoded by the *nsf*^*st*53 ^allele. Clearly, at least some functional NSF is required for survival as evidenced by the fact that co-injection of *nsf*-MO and *nsfb*-MO into *nsf*^*st*53 ^embryos was lethal (Additional file 1 Table S1). The persistence of *gnrh3 *in immature, pre-migratory *nsf*^*st*53 ^neurons, coupled with the ability to dampen *oxtl *transcripts following an acute salt challenge in wild-type zebrafish, implies that sub-populations of neuroendocrine cells escape the consequences of disrupted NSF function. Thus, we speculate that increased physiological demand and secretion of neuropeptide cargo during the embryo-to-larva transition accounts for this late-stage loss of neuroendocrine transcripts in *nsf *mutants.

The neuroendocrine phenotype exhibited by the *nsf*^*st*53 ^zebrafish is partially mimicked in *munc18-1 *mutant mice, which exhibit a selective and stage-dependent (E18) decrease primarily in neuropeptide transcript expression [41]. Munc18-1 is essential for regulated exocytosis. The authors of this study posited that transcripts encoding 'consumables' such as secreted products are selectively downregulated in the *munc18-1 *synaptically silenced neurons. Although neuropeptide protein levels and localization were not assessed in their study, our findings predict that neuropeptides would accumulate in *munc18-1 *hypothalami. We can now extend their hypothesis by suggesting that unspent neuropeptide consumables feed back to silence neuroendocrine transcriptional programs, as shown in our model figure (Figure 8).

Our study is now the second to identify *nsf *as essential in a specific neuronal cell-type program using a forward genetic screen in zebrafish. Previously, Talbot and colleagues [34] unexpectedly found *nsf *to be essential for organization of myelinated axons, with mutant zebrafish exhibiting decreased myelin basic protein expression and altered sodium channel clustering. Here, we find that this ubiquitous protein is also essential for maintenance of neuroendocrine transcriptional programs. These distinct phenotypes illustrate how different neuronal populations adjust to deficits in membrane fusion and intracellular trafficking. We suggest that hypothalamic neurons mount a stage-dependent adaptive response to this major secretory defect by silencing transcriptional programs. For other neuronal cell types, loss of *nsf *results in vastly different cellular responses. Indeed, we observed prominent cell death in many sensory organs, including photoreceptors, olfactory sensory neurons, and mechanosensory cells in *nsf *mutants. By contrast, no measurable cell death was observed in *nsf *mutant hypothalami as evidenced by the normal gross organization of the *nsf*^*st*53 ^hypothalamus, normal HuC/D staining, which marks early neuronal differentiation, and normal staining with the SV2 pan-neuronal vesicle marker. Targeted knockdown of *nsf *or *nsfb *in the mutant background also ruled out that survival of hypothalamic cells in *nsf*^*st*53 ^was due to compensation by the duplicate gene *nsfb *and/or maternally deposited *nsf *mRNA. Taken together, hypothalamic neurons, unlike other neuronal populations, are able to survive and maintain normal cellular integrity in the face of this major secretory deficit.

The phenotypes observed in *nsf*^*st*53 ^hypothalamic neurons, including the complete loss of neuroendocrine transcripts and the accumulation of ER-like membranes, are reminiscent of, but different from, other well-described secretory feedback signaling pathways. Regulation of nuclear targets by ER-associated events are well established for unfolded protein responsive (UPR) genes [42] and for sterol regulatory genes (reviewed in [43]). Here, we have yet to identify the precise molecular nature of the feedback signal; whether these signals arise from the buildup of ER-like membranes, from accumulation of neuropeptides in the cytoplasm, or from the inability to secrete neuropeptides at the plasma membrane remains to be determined. The build up of ER-like membrane in the cytoplasm of *nsf *hypothalamic neurons suggests that neuropeptidergic cells are responsive to a loss of secretion and not a general trafficking defect. Indeed, we can mimic this phenotype with a transient block on secretion in wild-type animals with botox injections. Thus, it is possible that ER-to-golgi transport in *nsf*^*st*53 ^neurons is partially intact, but becomes overloaded when functional demands are placed on these neurosecretory neurons in the embryo-to-lava transition.

This cytoplasmic-to-nuclear signaling hypothesis assumes that phenotypes observed in *nsf*^*st*53 ^hypothalami are cell-autonomous, as documented previously [34]. In our case blastomere transplantation studies into the hypothalamus are technically challenging because transplanted clone sizes in this brain region are small, and because selection of the correct *in situ *hybridization (ISH) probe, each recognizing a very small number of cells, is difficult. Despite these inherent limitations, preliminary transplantation data revealed expression of neuroendocrine markers in the medial hypothalamus of wild-type → *nsf*^*st*53 ^chimeras (data not shown), supporting the notion that similar to other neurons, NSF functions autonomously in specialized neuroendocrine neurons.

## Conclusion

The unexpected role of *nsf *in maintenance of hypothalamic cell type markers at late developmental stages implies that hypothalamic neurons adapt to and survive a major trafficking defect by modifying their transcriptional programs. We speculate that unlike sensory neurons, which undergo apoptosis, hypothalamic neurons cope with this major cellular stress because these ancient and evolutionarily conserved neurons are inherently programmed to defend and maintain homeostatic physiological responses when confronted with changing environments.

## Materials and methods

### Zebrafish

All research involving zebrafish was approved and carried out according to guidelines of the UCSF IACUC committee. Zebrafish were maintained at 28°C under 14 h light, 10 h dark as described previously [27]. *Nsf*^*st*25 ^and *nsf*^*st*53 ^zebrafish were kind gifts from W Talbot (Stanford University). Adult *nsf*^+/- ^zebrafish were fed a mixture of fish flakes and brine shrimp twice per day. The 5 dpf fish were maintained in egg water, consisting of 0.03% Instant Ocean→∀ sea salts (Spectrum Brands, Atlanta, GA, USA) in distilled water and not supplemented with any food. Embryos were obtained by natural crosses of identified parents and zebrafish embryo and larvae stages were determined according to [44]. The VBA phenotype was assayed by placing 5 dpf zebrafish on a white background for >15 minutes. Fish that remained dark in color and failed to have inflated swim bladders were selected as mutant zebrafish. Zebrafish <5 dpf were genotyped as previously reported in [34].

### Length measurements

Mutant and wild-type zebrafish (n = 10 each) were sorted for their dark phenotype at 5 dpf and then placed on ice to paralyze them but were kept alive. They were then transferred to a 3% agarose mold and their images were captured under an Olympus light microscope equipped with a CCD camera (Center Valley, PA, USA). The zebrafish in each image was measured from nose to tip of the tail to the nearest 0.1 cm using a standard ruler and the scale was adjusted appropriately using the scale bar present within the captured image. Statistical analyses were conducted by unpaired *t*-test.

### Whole mount in *situ *hybridization

Mutant and wild-type embryos/larvae were fixed in 4% paraformaldehyde (PFA) in phosphate-buffered saline (PBS) overnight at 4°C and transferred to 100% MeOH and stored at -20°C until use. Embryos were rehydrated into PBS-Tween (PBS-Tw; 0.1% Tween-20), bleached (72 hpf to 5 dpf; 1% H_2_O_2 _+ 5% formamide in PBS-Tw; 1 h), treated with proteinase K (20 μg/ml; 8 minutes), refixed (4% PFA; 20 minutes), prehybridized (65 to 70°C; 1 h) and hybridized (65 to 70°C; overnight) with hypothalamic riboprobes. Embryos were washed (50% formamide/50% 2× SSC; 2× SSC; 0.2× SSC), blocked (5% sheep serum heat inactive) and incubated overnight at 4°C with α-digoxigenin (DIG) antibody followed by washing and a second incubation with p-nitroblue tetrazolium chloride/5-bromo-4-chloro-3-indolyl phosphate (NBT/BCIP) in staining buffer (100 mM NaCl, 50 mM MgCl_2_, 100 mM Tris-HCl, 0.1% Tween 20). The color reaction was monitored for several hours and stopped by washing with PBS-Tw. The embryos were re-fixed and transferred to 90% glycerol for clearing. Embryos were captured using an Axiocam camera (Carl Zeiss, Thornwood, NY, USA). TUNEL-based cell death assays were carried out using this procedure with minor revisions according to manufacturer's protocol (ApopTag; Millipore Corporation, Billerica, MA, USA). DIG-cRNA probes were synthesized (Roche, Indianapolis, IN, USA) from the following generously gifted plasmids: *pomc*, Roger Cone (Vanderbilt University Medical Center); *hcrt*, Alexander Schier (Harvard University); *nsf*, William Talbot (Stanford); *oxtl *and *avpl*, Eric Glasgow (Georgetown University); *npy*, Peter Schoonheim (UCSF); *emx1*, *Su Guo *(UCSF); *crh*, Limor Ziv (UCSF); *ff1d*, Miyuki Suzawa (UCSF); *nkx2.1a*, Steve Wilson (University College London); g*nrh3*, Yonathan Zohar (Cambridge University).

### Immunohistochemistry on sectioned zebrafish

Mutant and wild-type larvae were fixed in 4% PFA/PBS overnight at 4°C and cryoprotected in 30% sucrose/PBS overnight at 4°C. For horizontal sections, zebrafish were embedded in OCT (Tissue-Tek, Sakura Finetek, Tokyo, Japan) and sectioned at 12 μm (Leica Microsystems, Bannockburn, IL, USA). Sectioned zebrafish were left to dry (overnight at 25°C), then washed in PBS-Tw, incubated in block (10% Normal sheep serum/PBS; 1 h at 25°C), and left to incubate overnight with α-HuC/D (1:400; Invitrogen, Carlsbad, CA, USA), α-SV2 (1:50; Developmental Studies Hybridoma Bank, University of Iowa), α-hypocretin (1:500; Dr Kohgo, Asahikawa Medical College, Japan), α-GnRH (1:1,500; Gunma University, Japan), α-Tyrosine Hydroxylase (1:100; Chemicon, Temecula, CA, USA) and α-NPY (1:1,000; Dr Jo Harrold, University of Liverpool). After washing in PBS-Tw, sections were incubated with Alexa-488 (1:200; Invitrogen) for 4 h, 25°C. Prior to mounting, sections were stained using Hoechst 33258 (Invitrogen). Confocal images were captured using a Zeiss LSM 5 Pascal microscope and software. Confocal stacks were further processed using ImageJ software. Z-projections of a few slices were made and comparisons were made between images that were processed equivalently. Fluorescence images were adjusted in Adobe Photoshop using the brightness/contrast, levels, and curves functions in order to best represent the protein levels.

### Real-time quantitative PCR

Real-time quantitative PCR was performed as described previously [45]. The head was dissected from the tail just prior to the swim bladder of 5 dpf mutant and wild-type zebrafish and the RNA was isolated from both head and tail using TrizolT. All data are normalized to one wild-type 'head' sample (taken to be 1) and the error bar represents the variation of the other 'head' samples (n = 4). Prior to routine use, all primer sets (Primer Express v2.0, Applied Biosystems, Foster City, CA, USA) were validated to ensure amplification of a single product with appropriate efficiency. Data obtained from the PCR reaction were analyzed using the comparative C_T _method (User Bulletin No. 2, PerkinElmer Life Sciences, Waltham, MA USA). Sequences are provided in Additional file 1 (Table S2).

### Osmolar challenge

For high salt, osmolar challenge, 4-dpf larvae were transferred to egg water composed of either 0.3% Instant Ocean→∀ (challenge) or 0.03% Instant Ocean→∀ (control) and maintained at 28°C. Larvae were collected 24 h later and fixed immediately using cold 4% PFA.

### Morpholino analyses

MO analyses were conducted as published previously [5]. Briefly, MOs were designed to bind sequences surrounding the initiating methionine (Gene Tools, Philomath, OR, USA). MOs were resuspended in water to a working solution of 1 μM and injected into the yolk of one- to four-cell stage embryos (n = 100 embryos/MO; repeated 3 times). Effective doses were determined separately for each MO. Larvae (5 dpf) were collected, fixed overnight and stored in 100% MeOH at 20°C until whole mount ISH was performed. Sequences are provided in Table S2 (Additional file 1).

### Electron microscopy

Tissue was fixed in 2% glutaraldehyde, 1% paraformaldehyde in 0.1 M sodium cacodylate buffer, pH 7.4, postfixed in 2% osmium tetroxide in the same buffer, and block stained in 2% aqueous uranyl acetate, dehydrated, infiltrated, and embedded in LX-112 resin (Ladd Research Industries, Burlington, VT, USA). Toluidine blue stained semi-thin sections were made to locate the area of interest, using the eyes as a guideline. Samples were ultrathin sectioned on a Reichert-Jung (Leica) Ultracut S ultramicrotome (Leica Microsystems, Bannockburn, IL USA), and stained with 0.8% lead citrate. Grids were examined on a JEOL JEM-1230 transmission electron microscope (JEOL USA, Inc., Peabody, MA, USA) and photographed using the Gatan Ultrascan 1000 digital camera (Gatan Inc., Warrendale, PA, USA).

### Botulinum toxin analyses

Botulinum toxin injections and analyses were conducted as published previously [46]. Briefly, progeny from in-crosses of heterozygous *nsf*^53 ^adults were injected at the one-cell stage with an approximately 5 nl bolus of 0.1 to 1.0 ng/nl BtTxB (EMDBiosciences, Darmstadt, Germany). Larvae (5 dpf) that lacked a startle response were placed either into fix (4% PFA, overnight, 16°C) or Trizol™ (-80°C) until processed for ISH and quantitative PCR, respectively.

## Abbreviations

dpf: days post-fertilization; E: embryonic day; EM: electron microscopy; ER: endoplasmic reticulum; GnRH: gonadotropin-releasing hormone; hpf: hours post-fertilization; ISH: *in situ *hybridization; MO: morpholino oligonucleotide; NPY: neuropeptide Y; NSF: N-ethylmaleimide sensitive factor; *oxtl*: *oxytocin-like*; avpl: arginine vasopressin-like; PBS: phosphate-buffered saline; PBS-Tw: PBS Tween; PFA: paraformaldehyde; *pomc*: *proopiomelanocortin*; SV2: synaptic vesicle protein 2; TUNEL: Terminal deoxynucleotidyltransferase-mediated dUTP nick end labeling; VBA: visually mediated background adaptation.

## Competing interests

The authors declare that they have no competing interests.

## Authors' contributions

DMK conducted the initial characterization of *nsf *mutant zebrafish including analysis of transcripts, proteins, TUNEL, morpholino studies, and EM images, and drafted the manuscript. LMN injected botulinum toxin B and aided in confocal microscopy. JSW carried out the EM experiments. HB participated in the design of the study, manuscript preparation, and provided invaluable assistance throughout the project. HAI conceived of and participated in the design and coordination of the study, as well as manuscript preparation.

## Supplementary Material

Additional file 1 **Sup1**. Supplemental figures S1–S5, and supplemental tables 1 and 2. Click here for file
