# Supplementary material for: Neuroendocrine transcriptional programs adapt dynamically to the supply and demand for neuropeptides as revealed in NSF mutant zebrafish
Source: Neural Dev. 2009 Jun 23;4:22. doi: 10.1186/1749-8104-4-22 (PMC2715394; doi:10.1186/1749-8104-4-22)
Supplement: Additional file 1 — Sup1. Supplemental figures S1–S5, and supplemental tables 1 and 2. [file 1749-8104-4-22-S1.pdf]

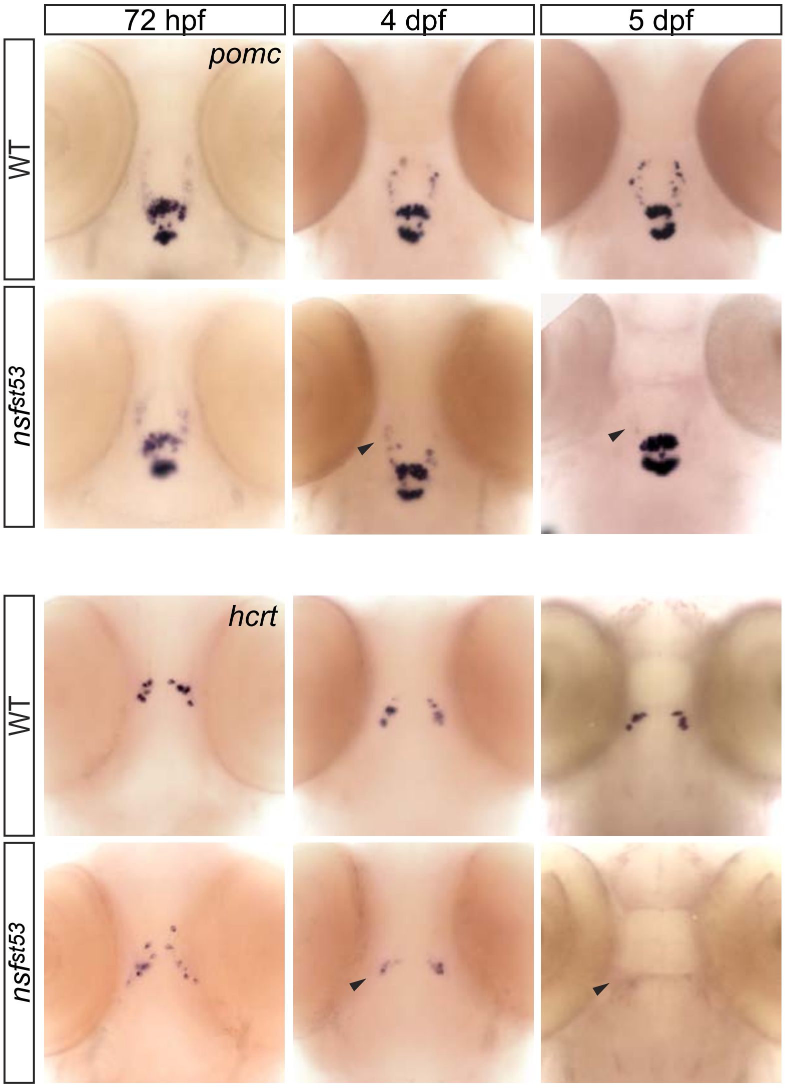

Kurrasch et al, Figure S1. Loss of hypothalamic markers occurs during embryo-to-larva transition. Whole-mount expression patterns are shown for *pomc* (top panels) and *hcrt* (bottom panels) in 72hpf, 4dpf, and 5dpf WT and *nsf*^{st53} zebrafish (n≥15 per timepoint). Progressive loss of transcripts are noted (black arrowheads).

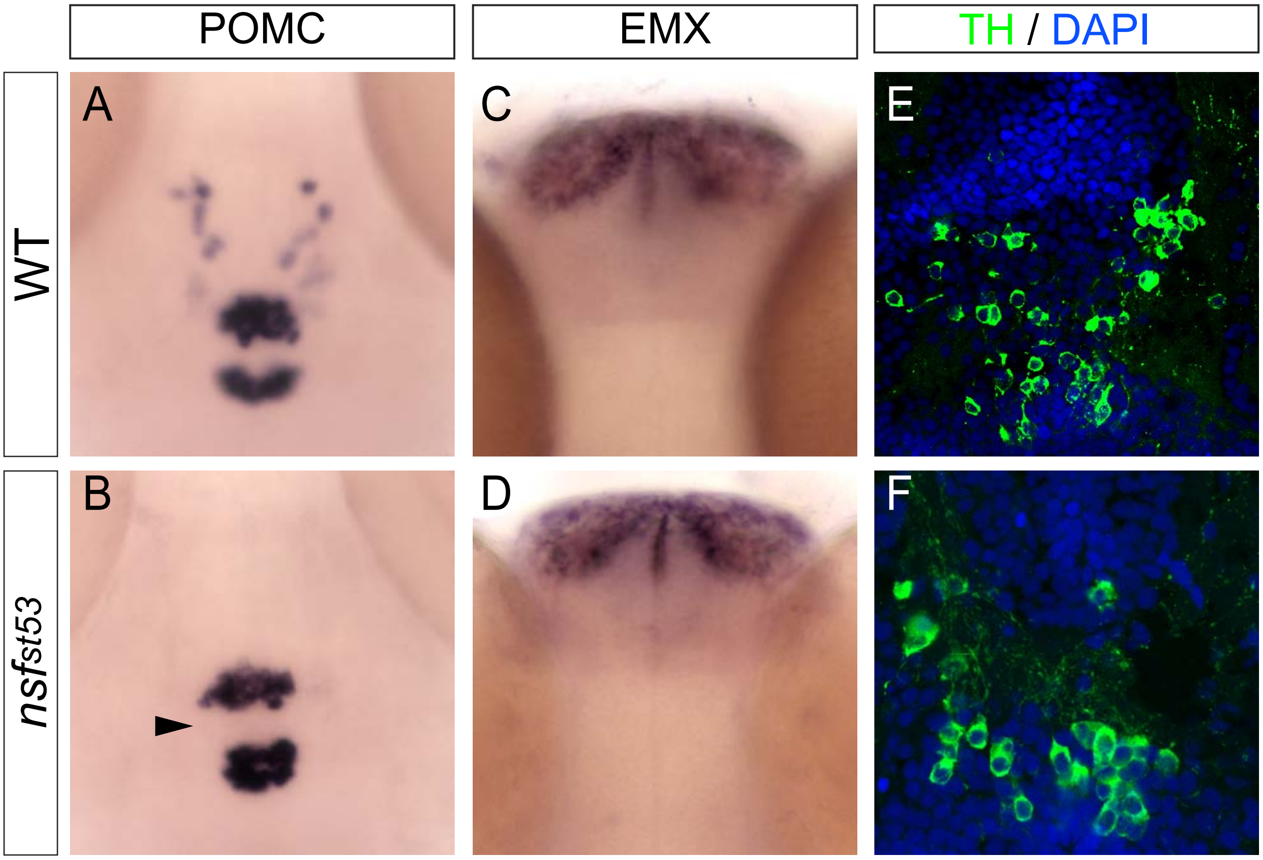

Kurrasch et al, Figure S2. Transcript and protein levels are equal in other brain regions (A-D) and in hypothalamic catecholamine neurons (E-F) in WT and *nsf* mutant 5dpf larvae. (A-D) Whole-mount expression patterns are shown for *pomc* (A-B) and *emx* (C-D) in WT and *nsf*^{st53} zebrafish (n≥8 per marker per genotype). Maintained expression of *pomc* in *nsf* mutant pituitary is noted (black arrowhead). (E-F) Tyrosine hydroxylase protein levels in hypothalamic neurons is shown in sectioned WT and *nsf*^{st53} zebrafish (n=7 ea). Cell bodies are shown with DAPI stain.

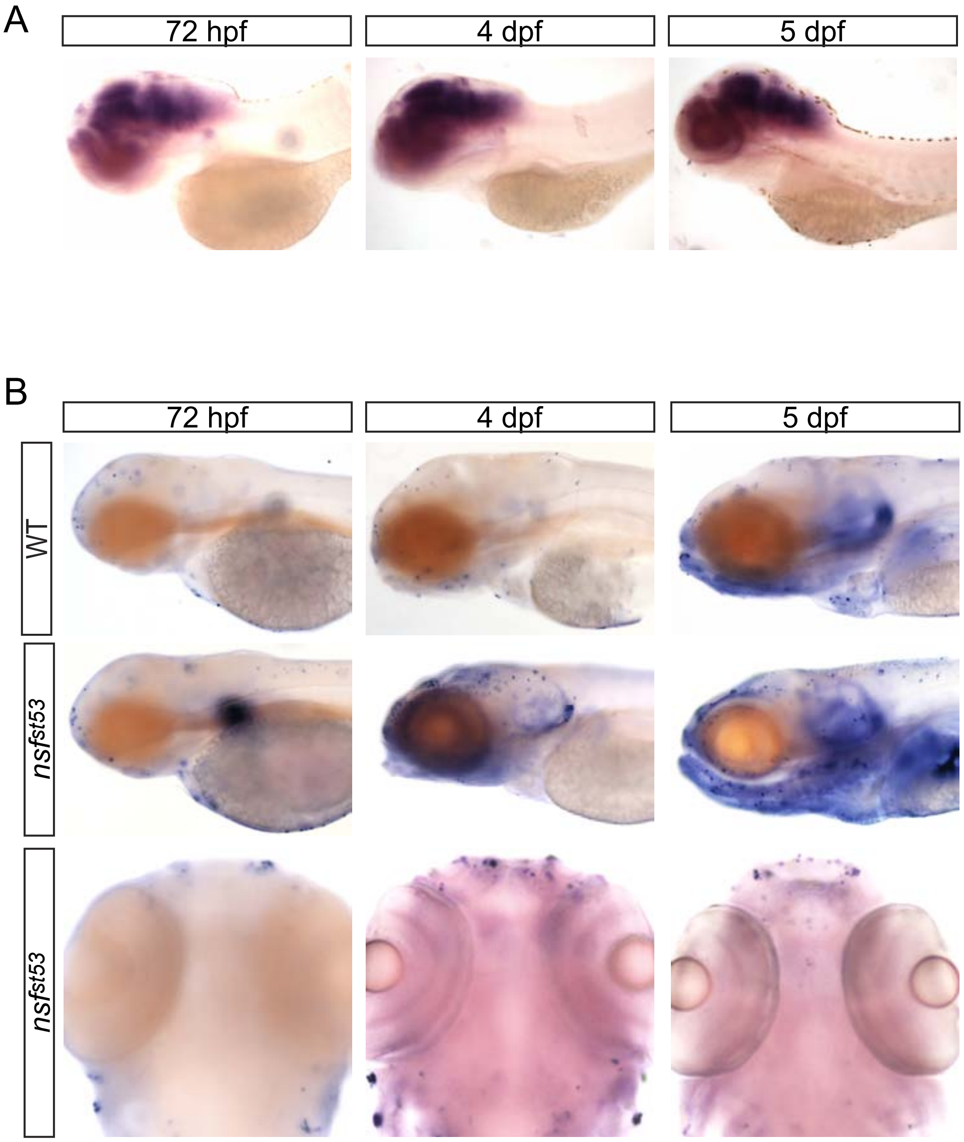

Kurrasch et al, Figure S3. *nsf*^{st53} hypothalamic neurons do not appear to undergo apoptosis. (A) Lateral view of a whole-mount ISH for *nsf* in WT zebrafish during the embryo-to-larva transition is shown. (B) Apoptosis in wild type and *nsf*^{st53} zebrafish (lateral view, middle panels; dorsal view at the hypothalamus, bottom panels) fixed at 24 hour intervals during the embryo-to-larva transition is shown. Images are representative of at least two experiments; n=5 per genotype per timepoint.

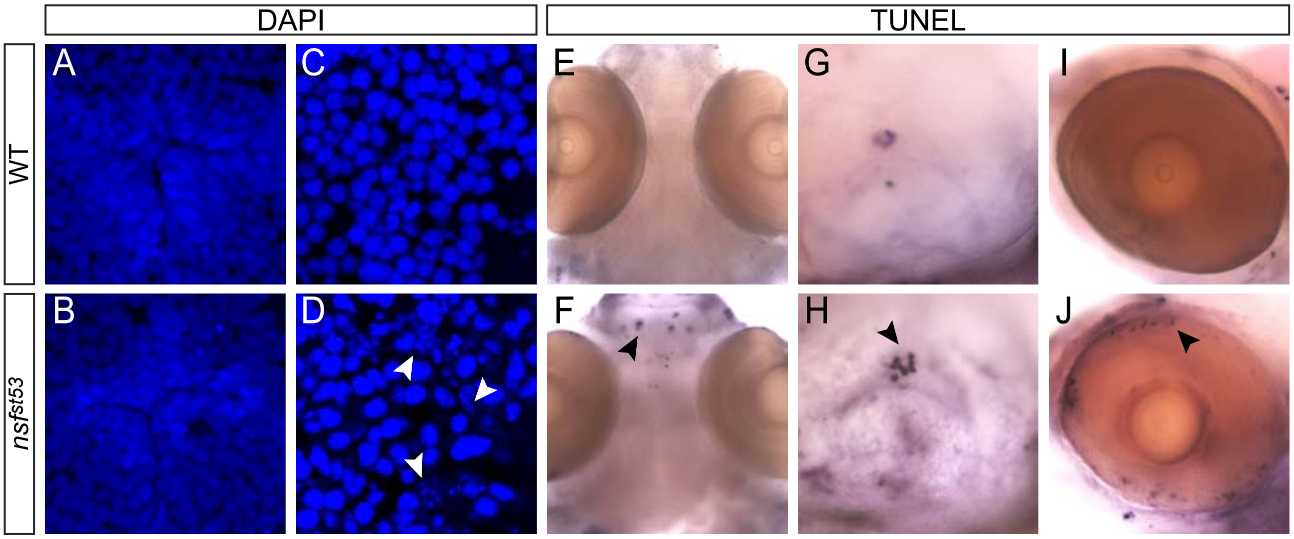

Kurrasch et al, Figure S4. Cell death is observed in sensory neurons in *nsf* mutant larvae. DAPI stain in the hypothalamus (A, B) and the posterior tuberculum of wild type and *nsf*^{st53} sectioned (12 μm horizontal sections) 5dpf zebrafish is shown. Morphological changes associated with cell death, such as cell asymmetry and chromatin condensation is noted (white arrowheads; D). Cell death is not observed in the hypothalamus (A,B), but is observed in olfactory terminals (F), otic capsule (H) and photoreceptor cells (J) of mutant zebrafish. Images representative of duplicate experiments, n≥5 per genotype.

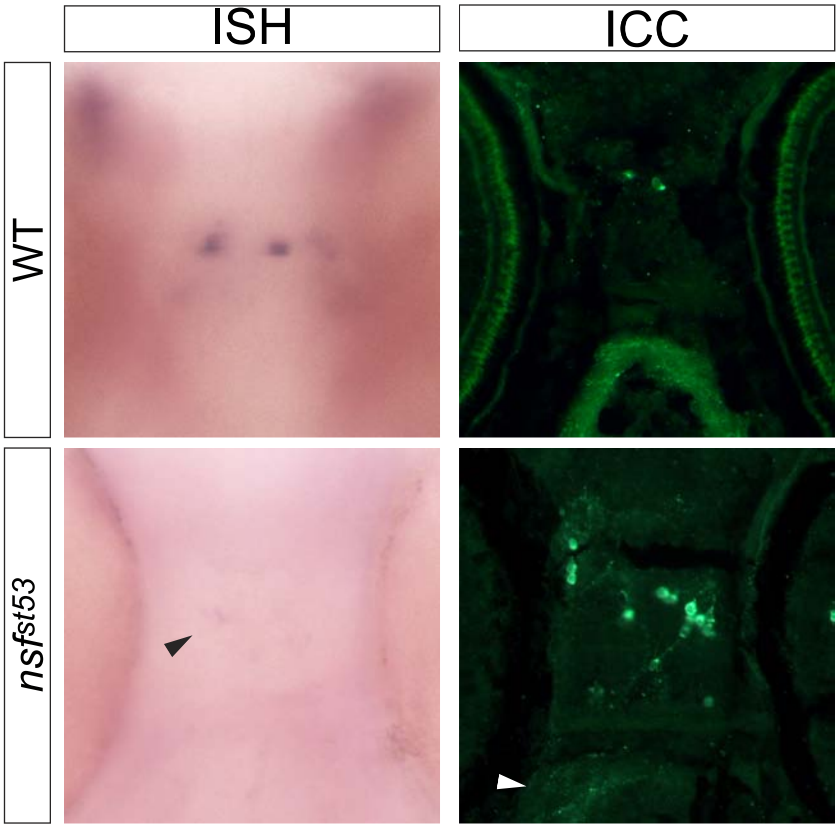

Kurrasch et al, Figure S5. *npy* transcripts are absent but NPY protein is accumulated in *nsf* mutant larvae. Whole-mount staining for *npy* expression and immunocytochemistry for NPY protein in wild type and *nsf*^{st53} 5dpf zebrafish is shown (n=5 per experiment per genotype). Loss of transcript (black arrow-head) is noted. The contrast between the decreased NPY levels in peripheral projections (white arrowhead) but accumulation of NPY in the soma of *nsf* mutant zebrafish and the levels observed in WT is highlighted.

Kurrasch et al, Supplemental Table 1: Summary of morpholino studies

Genotype	Morpholino	Question Addressed	Gross Phenotype	ISH Phenotype
nsf^{st53}	nsf	Role of maternal contributions	Same as *nsf*^{st53}; Dark, longer, paralyzed	Slight decrease oxtl and GnRH expression
nsf^{st53}	nsf-b	Functional compensation	Same as *nsf*^{st53}; Dark, longer, paralyzed	No effect oxtl and GnRH expression
nsf^{st53}	nsf + nsf-b	Requirement of NSF	Early death; 2/50 embryos viable	N/A
WT	nsf	Efficacy of MO	Same as *nsf*^{st53}; Dark, paralyzed	N/A
WT	nsf-b	Efficacy of MO	No effect; WT phenotype	N/A
WT	nsf + nsf-b	Efficacy of MO	Some death, 30/50 embryos viable	N/A

Information pertaining to the genotype, morpholino injected, and resulting phenotype is given. N/A, ISH was not performed. See Supplemental Table 2 for morpholino sequences.

Kurrasch et al, Supplemental Table 2. Primer sequences for zebrafish morpholino and qPCR

MORPHOLINO

Symbol	Gene	Accession #	Target Region	Sequence (5'-3')
nsf	N-ethylmaleimide-sensitive factor	NM_001044328	ATG	CGTCTGCACACAGACAAATAGTAAT
Nsf-b	N-ethylmaleimide-sensitive factor-b	NM_001024454	ATG	GCAAAGGATTGATGAAATGGTAGCA

qPCR PRIMER SETS

Symbol	Gene	Accession #	Forward Primer (5'-3')	Reverse Primer (5'-3')
nsf	N-ethylmaleimide-sensitive factor	NM_001044328	TCCTCGTCATCGGCATGA	TCAAATCGGCCAGGTCTCA
Nsf-b	N-ethylmaleimide-sensitive factor-b	NM_001024454	GGAGCTTCTTGGAGGCTTTCA	CGCTCTGTCCTTTCACCTGTT
oxtl	oxytocin-like, isotocin neurophysin	NM_178291	GCTGTTTCGGCCCCAGTA	GTTTCTGGAGAGCCGACCAA
nr5a1b	nuclear receptor subfamily 5, group A, member 1b	NM_212834	GCGAGACCGGGCGTTAA	CCACTGGCCCGGATG
avpl	arginine vasopressin-like, vasotocin neurophysin	NM_178293	TCGTCTGCCTGCTACATCCA	TCCGGCTGGGATCTCTTG
crh	corticotropin releasing hormone	NM_001007379	TCCGGCTCGGCAACA	AAGCTGTCGGCTGGAGATC
hcrt	hypocretin (orexin) neuropeptide precursor	NM_001077392	CCGAGCAGGACGCAGAA	GTCGTTGTTGAGATGCACTAAATGT
	cyclophilin	NM_212758	TCACACTGAAACACGGAGGCA	GCTTACCGTCCAGCCAGTTG

Symbol, common name and accession number are given, as well as the 5'-3' oligo sequence. Morpholinos were ordered directly through Gene Tools, LLC (Philomath, OR). Target region refers to where translation was blocked. ATG refers to morpholinos that span the start site.
